# Shellac‐Mediated Assembly of Nanoparticles for mRNA Delivery

**DOI:** 10.1002/adhm.202505918

**Published:** 2026-04-21

**Authors:** Meizhang Lu, Jingqu Chen, Zhixing Lin, Wanjun Xu, Tianzheng Wang, Shiyao Li, Die Yang, Frank Caruso

**Affiliations:** ^1^ Department of Chemical Engineering The University of Melbourne Parkville Victoria Australia

**Keywords:** biomedicine, gene delivery, oral administration, self‐assembly, supramolecular chemistry

## Abstract

Cationic polymers have been extensively explored for messenger RNA (mRNA) delivery owing to their ability to condense nucleic acids and facilitate cellular uptake. High‐molecular‐weight polymers typically achieve high transfection efficiency but exhibit substantial cytotoxicity, whereas low‐molecular‐weight polymers show reduced cytotoxicity but low transfection efficiency. To address the tradeoff between toxicity and transfection efficiency, herein, we present a simple and versatile shellac (SL)‐mediated assembly strategy, whereby mRNA is complexed with low‐molecular‐weight cationic polymers (e.g., polyethyleneimine) and subsequently stabilized by SL to form nanoparticles under mild conditions. The SL‐based nanoparticles exhibit >90% mRNA encapsulation efficiency and enable mRNA transfection across various cell types while showing negligible cytotoxicity. Furthermore, the SL‐based nanoparticle platform enables protein expression and gene editing in mice through both intravenous and oral administration routes. This work highlights the potential of SL as a key building block to direct the assembly and delivery of mRNA, enabling the rational design of modular and versatile nucleic acid delivery platforms for diverse biological applications.

## Graphical abstract

A shellac‐mediated nanoparticle assembly strategy is presented, involving the complexation of mRNA and low‐molecular‐weight cationic molecules (e.g., polyethyleneimine, chitosan, lipids, and poly‐l‐lysine), followed by capping with shellac. This nanoparticle platform enables mRNA transfection following intravenous injection and facilitates gene editing in the colon after oral administration.

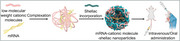

## 1 Introduction

Protein synthesis via messenger RNA (mRNA) translation highlights the central role of mRNA in the transfer of genetic information, positioning it as a promising platform for disease treatment and prevention [1]. The success of mRNA‐based COVID‐19 vaccines developed by Pfizer and Moderna further demonstrates the translational potential of mRNA technology in vaccine development and healthcare [2]. However, mRNA is susceptible to RNase‐mediated degradation, while its large molecular weight and highly anionic nature pose significant barriers to efficient intracellular delivery. These challenges have inspired the development of a wide range of nanoparticle (NP)‐based delivery platforms. Most NP platforms involve the incorporation of cationic, amine‐containing components (e.g., ionizable lipids [3], polyethyleneimine (PEI) [4], and poly‐l‐lysine (PLys) [5]) for enhancing mRNA delivery efficacy [3], mainly by promoting electrostatic interactions with mRNA and facilitating endosomal escape upon endocytosis. However, the molecular weight of cationic components can influence delivery efficacy and cytotoxicity [6]. High‐molecular‐weight cationic polymers typically offer good transfection efficiency, but often exhibit cytotoxicity and poor biocompatibility. In contrast, low‐molecular‐weight polymers show lower cytotoxicity, but typically lack sufficient transfection capability because of weaker mRNA binding or cellular uptake. For example, PEI with a high molecular weight (e.g., 25 kDa) can induce modest apoptosis, strong necrosis, and DNA damage [7, 8], whereas PEI with a low molecular weight (e.g., 1.8 kDa) exhibits substantially lower cytotoxicity but a compromised transfection efficiency (< 5%) [9, 10]. Therefore, NPs need to be appropriately engineered to balance the trade‐off between transfection efficiency and toxicity. A common strategy to improve the transfection efficiency of low‐molecular‐weight PEIs involves conjugating them with highly biocompatible or hydrophobic moieties to enhance subsequent mRNA complexation and facilitate membrane interactions [11, 12]. For example, a cyclodextrin‐modified 2 kDa PEI platform demonstrated mRNA delivery efficacy. Specifically, the modification reduced toxicity and resulted in a higher transfection efficiency [13]. Similarly, a vitamin E succinate‐modified PEI copolymer was developed to deliver mRNA vaccines, eliciting strong humoral and cellular immune responses following intramuscular injection [9]. However, despite advances, current approaches are limited by the need for sophisticated synthetic procedures. Thus, developing a simple and effective method for preparing mRNA NPs that can achieve both high transfection efficiency and low cytotoxicity is still lacking.

Shellac (SL), a natural resin excreted by the insect *Laccifer lacca*, is generally recognized as safe by the U.S. Food and Drug Administration [14, 15]. It is composed of aleuritic acid and cyclic terpene acids connected by ester bonds, which act as the hydrophobic and hydrophilic segments, respectively [16, 17]. Owing to its amphiphilic property [18], SL is expected to facilitate the formation of compact NPs with mRNA and low‐molecular‐weight cationic polymers, improving NP stability and facilitating membrane interactions, thereby increasing transfection efficiency. We hypothesized that the versatile supramolecular binding affinity of SL, its neutral‐to‐negative charge that enables cationic shielding, and its ability to protect labile cargo from enzymatic degradation may provide a generalizable pathway for mRNA assembly and delivery.

**SCHEME 1:**
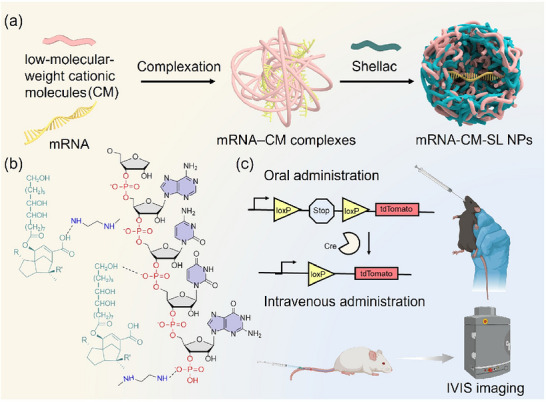
(a) Assembly and (b) possible interactions of mRNA‐CM‐SL NPs for (c) mRNA delivery in vivo by oral and intravenous administration.

In the present study, we report a facile SL‐mediated approach to assemble and stabilize mRNA within NPs under ambient conditions. In this approach, mRNA is first complexed with select cationic molecules (CMs), encompassing low‐molecular‐weight polymers (e.g., PEI), polysaccharides (e.g., chitosan), lipids (e.g., SM‐102), and PLys, followed by capping of the complexes with SL to yield mRNA‐loaded‐cationic molecule‐shellac NPs (i.e., mRNA‐CM‐SL NPs; Scheme 1). The electrostatic interactions conferred by the cationic motifs, combined with the versatile binding affinity of SL, afford over 90% mRNA encapsulation efficiency and mRNA transfection both in vitro and in mice. Notably, the pH‐dependent solubility of SL confers stability to the mRNA‐CM‐SL NPs in gastric fluids, allowing for deposition throughout the gastrointestinal (GI) tract and gene editing in the colon post‐oral gavage. Furthermore, cation shielding by SL preserves the viability of cells treated with these NPs. This study is expected to underpin the rational design of therapeutic delivery platforms using naturally occurring building blocks and, in particular, provides a versatile platform for mRNA delivery via both intravenous and oral administration, thus expanding its potential clinical applications.

## 2 Results and Discussion

**FIGURE 1:**
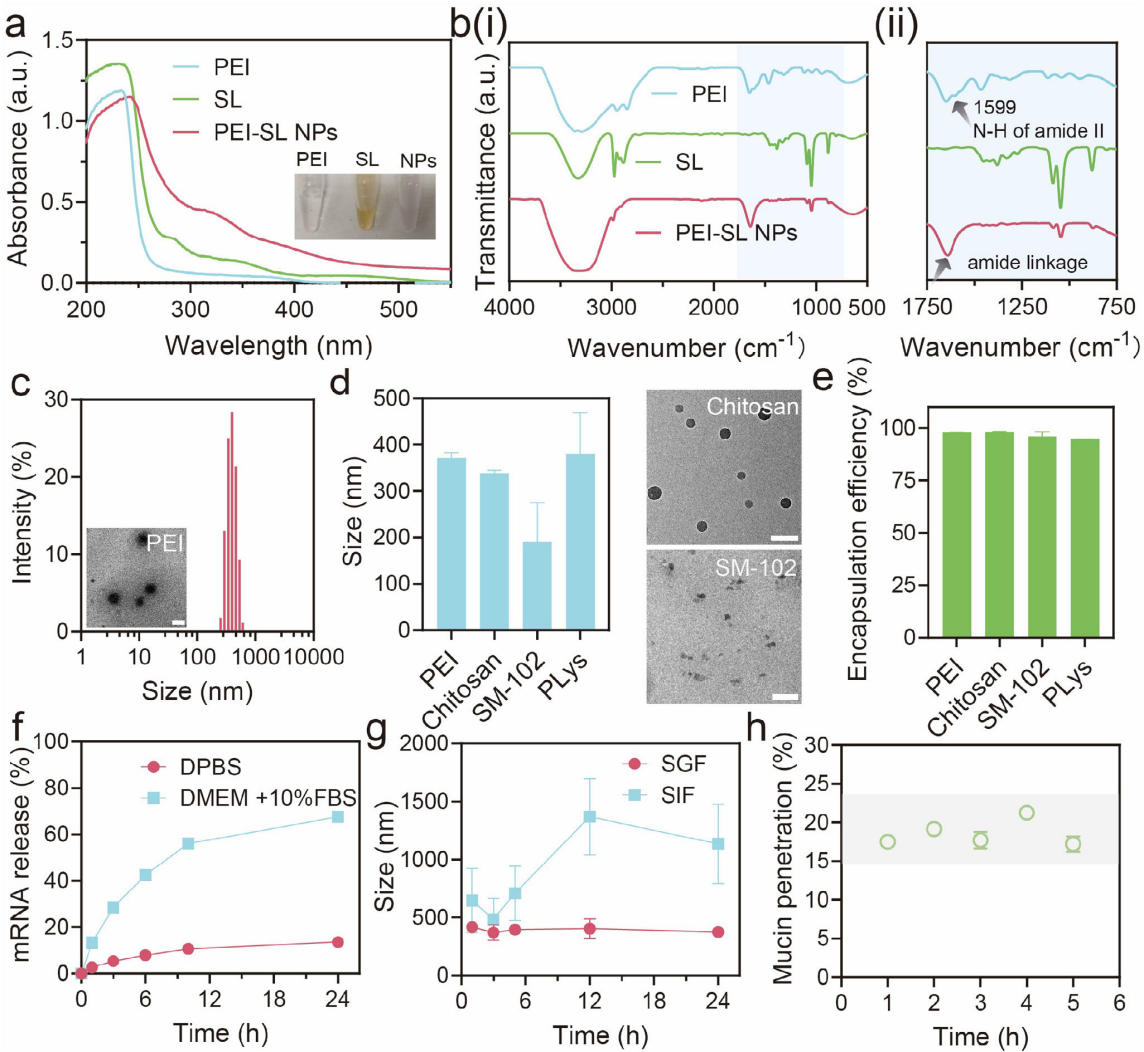
Characterization and versatility of mRNA‐CM‐SL NPs. (a) UV–vis spectra of PEI, SL, and PEI‐SL NPs. The inset shows solution color before and after mixing of PEI and SL. (b) Fourier transform infrared spectra of PEI, SL, and PEI‐SL NPs in the range of 4000–500 cm^−1^ (i) and 1750–750 cm^−1^ (ii). (c–e) mRNA‐CM‐SL NPs assembled using different CMs (PEI, chitosan, SM‐102, PLys). (c) Size distribution and representative transmission electron microscopy image of mRNA‐PEI‐SL NPs; scale bar is 200 nm. (d) Size and representative transmission electron microscopy images of mRNA‐CM‐SL NPs assembled from SL, mRNA, and various CMs; scale bars are 200 nm. (e) mRNA encapsulation efficiency of mRNA‐CM‐SL NPs assembled from various CMs. (f) Release of mRNA from mRNA‐PEI‐SL NPs in DPBS or DMEM+10% FBS at 37°C. (g) Changes in NP size of mRNA‐PEI‐SL NPs incubated in SGF and SIF for 24 h. (h) Mucin penetration efficiency of mRNA‐PEI‐SL NPs as a function of incubation time. In (d), (e), (g), and (h), data are presented as mean ± standard deviation (SD).

PEI‐SL NPs were assembled by dissolving SL in ethanol and subsequent mixing with low‐molecular‐weight PEI (e.g., *M*_w_ = 800 Da; PEI_800_) in water. The color transition of the original yellow solution to a pink suspension upon mixing, accompanied by a shift in the UV–vis absorbance band from 279 to 308 nm, indicates potential interactions between SL and PEI (Figure 1a). Furthermore, the absence of the band at 1599 cm^−1^ (ascribed to amide II in PEI) and the emergence of a sharp peak at 1639 cm^−1^ (ascribed to amide linkage (─CONH─)) in the Fourier transform infrared spectrum of PEI‐SL NPs indicate interactions between the carboxyl groups in SL and amine groups in PEI, suggesting the complexation of SL and PEI (Figure 1b).

Three types of mRNA complexes were prepared: mRNA–PEI, mRNA–SL, and mRNA complexed with PEI and then capping with SL, to explore their potential for biomedical applications. Among the three complexes formed, the mRNA–PEI complexes were the largest and featured the highest polydispersity (i.e., size = 825 nm ± 33 nm, polydispersity index (PDI) = 0.40) (Figure S1). The average size of the mRNA–SL complexes was 187 nm ± 3 nm, and the PDI was 0.15. Complexing mRNA with both PEI and SL resulted in the formation of solid NPs (i.e., mRNA‐PEI‐SL NPs) with an average size of 372 ± 10 nm and PDI of 0.07. The reduced size and lower PDI suggest that the incorporation of SL in the mRNA–PEI complex facilitates the assembly of the NPs and improves their monodispersity. The incorporation of SL also led to a reduction in zeta‐potential (i.e., from 31 to 7 mV when the mass ratio of PEI‐to‐mRNA was 10), indicating cation shielding by SL (Figure S2). Furthermore, from the encapsulation efficiency results, we found that mRNA–SL complexes exhibited a relatively low encapsulation (15%), likely due to the combined effect of electrostatic repulsion, hydrogen bonding, and hydrophobic interactions. In contrast, the mRNA–PEI complexes and mRNA‐PEI‐SL NPs showed a higher encapsulation efficiency (98%; Figure S3), which was likely due to the strong electrostatic interactions between PEI and mRNA. Collectively, these results demonstrate that complexation with cationic moieties facilitates mRNA encapsulation, while the incorporation of SL enables the formation of stable, mRNA‐loaded supramolecular NPs. To further explore the versatility of the mRNA‐CM‐SL NPs, other CMs (i.e., chitosan, PLys, and SM‐102) were assembled with mRNA to form mRNA–CM complexes. These complexes displayed a broad size distribution with an average diameter of >200 nm (Figure S4). Upon incorporation of SL, the resultant mRNA‐CM‐SL NPs showed a relatively narrow size distribution and less positive zeta‐potential, indicating cationic shielding from SL (Figure 1c,d; Figures S5–S7). Similarly, the SL‐mediated NPs also showed high mRNA encapsulation efficiency (Figure 1e).

To evaluate the stability of the NPs, mRNA‐PEI‐SL NPs were incubated in RNase‐free water and Dulbecco's modified Eagle medium (DMEM) supplemented with 10% fetal bovine serum (FBS). As shown in Figure S8, the mRNA‐PEI‐SL NPs remained relatively stable in biological media for 1 week, which may be attributed to the formation of a protein corona on the NP surface. The mRNA release experiments revealed that less than 20% of mRNA was released from the mRNA‐PEI‐SL NPs in Dulbecco's phosphate‐buffered saline (DPBS) within 24 h, whereas 67% of mRNA was released in biological media, which was more than twice that released from the mRNA–PEI complexes (Figure 1f; Figure S9). To examine the interactions for the stability of the mRNA‐PEI‐SL NPs, the size of the mRNA‐PEI‐SL NPs was measured upon incubation with urea (hydrogen bond competitor), Tween 20 (hydrophobic competitor), or sodium chloride (NaCl; electrostatic competitor). Exposure to either urea or NaCl led to larger particles, indicating particle aggregation. However, the size of the NPs rapidly decreased to ∼10 nm within 10 min of incubation with Tween 20 (Figure S10), suggesting that hydrophobic interactions play a role in stabilizing the mRNA‐PEI‐SL NPs. Collectively, these results suggest that multiple interactions stabilize the mRNA‐PEI‐SL NPs.

We next assessed the stability of the mRNA‐PEI‐SL NPs under GI‐mimetic conditions. The mRNA‐PEI‐SL NPs remained stable in simulated gastric fluid (SGF), as deduced by negligible changes in their size and morphology (Figure 1g; Figure S11). This finding suggests the potential of the mRNA‐PEI‐SL NPs to protect encapsulated bioactive payloads from degradation by gastric fluids and enzymes during transit through the GI tract. In contrast, distinct changes in NP size were observed in simulated intestinal fluid (SIF), likely due to aggregation caused by electrostatic interactions between deprotonated SL and PEI. The mucus‐penetration ability of the NPs was evaluated using an agarose gel [19]. The results demonstrated that approximately 20% of the NPs penetrated through the mucus layer (Figure 1h). Dynamic light scattering revealed an increase in NP size after incubation with mucin, indicating interactions between the NPs and mucin (Figure S12).

**FIGURE 2:**
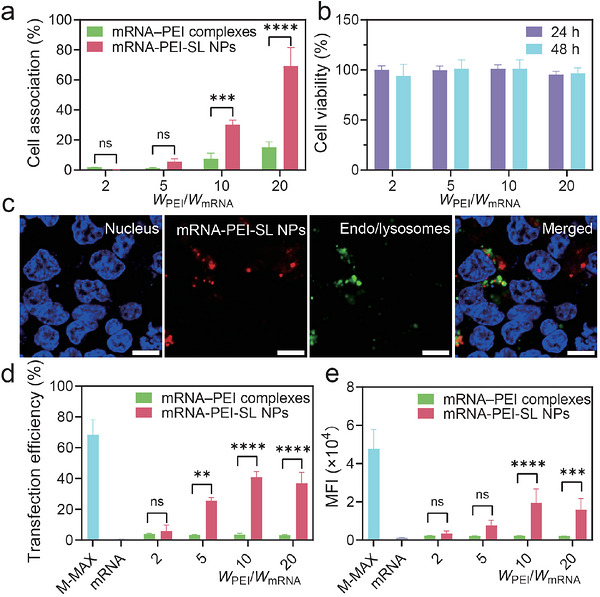
Biological interactions of mRNA‐PEI‐SL NPs. (a) Association of mRNA–PEI complexes and mRNA‐PEI‐SL NPs with HEK293T cells as a function of PEI‐to‐mRNA mass ratio at 6 h post‐incubation. (b) Viability of HEK293T cells after incubation with mRNA‐PEI‐SL NPs at different PEI‐to‐mRNA mass ratios for 24 and 48 h. (c) Confocal laser scanning microscopy imaging of a LysoTracker colocalization assay performed in HEK293T cells after incubation for 12 h with mRNA‐PEI‐SL NPs. Scale bars are 10 µm. (d) Transfection efficiency and (e) MFI of mRNA–PEI complexes and mRNA‐PEI‐SL NPs in HEK293T cells. M‐MAX, MessengerMAX. All experiments were performed in triplicates (*n* = 3), and data are presented as mean values ± SD. Statistical significance was analyzed using two‐way ANOVA with Tukey's multiple comparisons test. ns, not significant; ^*^ *p* < 0.05, ^**^ *p* < 0.01, ^***^ *p* < 0.001, and ^****^ *p* < 0.0001.

To explore the potential of the mRNA‐PEI‐SL NPs for biomedical applications, we first conducted cellular association studies. As shown in Figure 2a, the incorporation of SL enhanced association with HEK293T cells. For instance, in the absence of SL, the mRNA–PEI complexes displayed <20% cell association at 6 h post‐incubation, even when the PEI‐to‐mRNA mass ratio (*W*_PEI_/*W*_mRNA_) increased to 20:1. In contrast, incorporating SL enabled significantly higher cellular association, achieving approximately 70% at 6 h. In addition, the mRNA‐PEI‐SL NPs exhibited time‐dependent cellular association behavior; negligible cellular association was observed at 1 h, whereas nearly 100% association was achieved at 24 h (Figure S13). To investigate the mechanism underlying the cellular uptake of mRNA‐PEI‐SL NPs, different types of endocytic inhibitors, including pitstop2, EIPA, filipin from *Streptomyces filipinensis*, and cytochalasin D, were incubated with cells prior to NP treatment. As shown in Figure S14, incubation with EIPA, filipin, and cytochalasin D reduced cellular uptake to approximately 77%, 78%, and 61%, respectively, suggesting that the cellular entry of these NPs was mediated by a combination of micropinocytosis, caveolae‐mediated endocytosis, and clathrin‐mediated endocytosis. The cytotoxicity of the mRNA‐PEI‐SL NPs was then evaluated as a function of PEI‐to‐mRNA mass ratio. As shown in Figure 2b, the mRNA‐PEI‐SL NPs displayed negligible cytotoxicity to HEK293T cells following 24 or 48 h incubation. In contrast, the viabilities of the cells incubated with PEI_0.8k_ and PEI_25k_ were 100% and 50%, respectively, at 20 µg mL^−1^ (Figure S15). To investigate the potential of the NPs for oral delivery, the viability of Caco‐2 cells (a human intestinal cell line, commonly used for oral drug delivery models [20]) and RAW264.7 cells (a murine macrophage cell line [21]) after treatment with mRNA‐PEI‐SL NPs were studied. For both cell lines, cell viabilities remained higher than 80% at a NP concentration of 80 µg mL^−1^, suggesting the compatibility of the NPs for intracellular delivery (Figure S16). We next examined the secretion of tumor necrosis factor‐α (TNF‐α) in blood following intravenous administration in mice. No significant increase in TNF‐α was detected in the mRNA‐PEI‐SL NPs group compared with the DPBS control (Figure S17), indicating that the NPs exhibit a low inflammatory response. As most NPs are likely to undergo the endo/lysosomal pathway after cell internalization [22], we next investigated the colocalization of the mRNA‐PEI‐SL NPs with endo/lysosomal compartments. The endo/lysosomes were stained with LysoTracker Green, while mRNA was labeled with cyanine 5 (red). The minimal colocalization of green and red fluorescence signals observed in the confocal laser scanning microscopy images, along with a low Pearson's correlation coefficient value of 0.425, indicated some degree of endosomal escape of the mRNA‐PEI‐SL NPs (Figure 2c; Figure S18).

The transfection efficiency of the mRNA‐PEI‐SL NPs was explored using mRNA that encodes a reporter protein (i.e., mCherry). Similar to the pattern observed for cellular association, the mRNA–PEI complexes did not significantly interact with cells, resulting in a negligible transfection efficiency, regardless of the PEI‐to‐mRNA ratio. In contrast, the incorporation of SL enabled successful mRNA transfection, as indicated by both flow cytometry and confocal laser scanning microscopy images (Figure 2d,e; Figure S19). This is likely due to the increased hydrophobicity imparted by SL that further stabilized the NPs [12, 23] and facilitated cellular association (Figure 2a). Furthermore, the successful intracellular expression of mRNA suggests some degree of integrity of mRNA released from mRNA‐PEI‐SL NPs. Notably, the level of mRNA transfection was modulated by varying the PEI‐to‐mRNA mass ratio: increasing this ratio up to 10:1 gradually increased the transfection efficiency and mCherry mean fluorescence intensity (MFI; i.e., the level of mCherry expression within the cells) from approximately 6% to 41% and from 3454 to 19585, respectively. Therefore, a PEI‐to‐mRNA mass ratio of 10 was employed for further experiments.

The transfection efficiency of the NPs after pre‐treatments with SGF and SIF was evaluated using Caco‐2 cells. As seen in Figure S20, the mRNA‐PEI‐SL NPs retained their mRNA transfection ability following exposure to simulated GI fluids, resulting in a 2.7‐fold higher MFI relative to untreated cells. Collectively, these results demonstrate the potential of the lead formulation (i.e., mRNA‐PEI‐SL NPs with a PEI‐to‐mRNA mass ratio of 10:1) for the oral delivery of mRNA. To improve reproducibility, reporting, and re‐analysis, this study conforms to the Minimum Information Reporting in Bio–Nano Experimental Literature (MIRIBEL) standard [24], and a companion checklist is provided in the Supporting Information.

**FIGURE 3:**
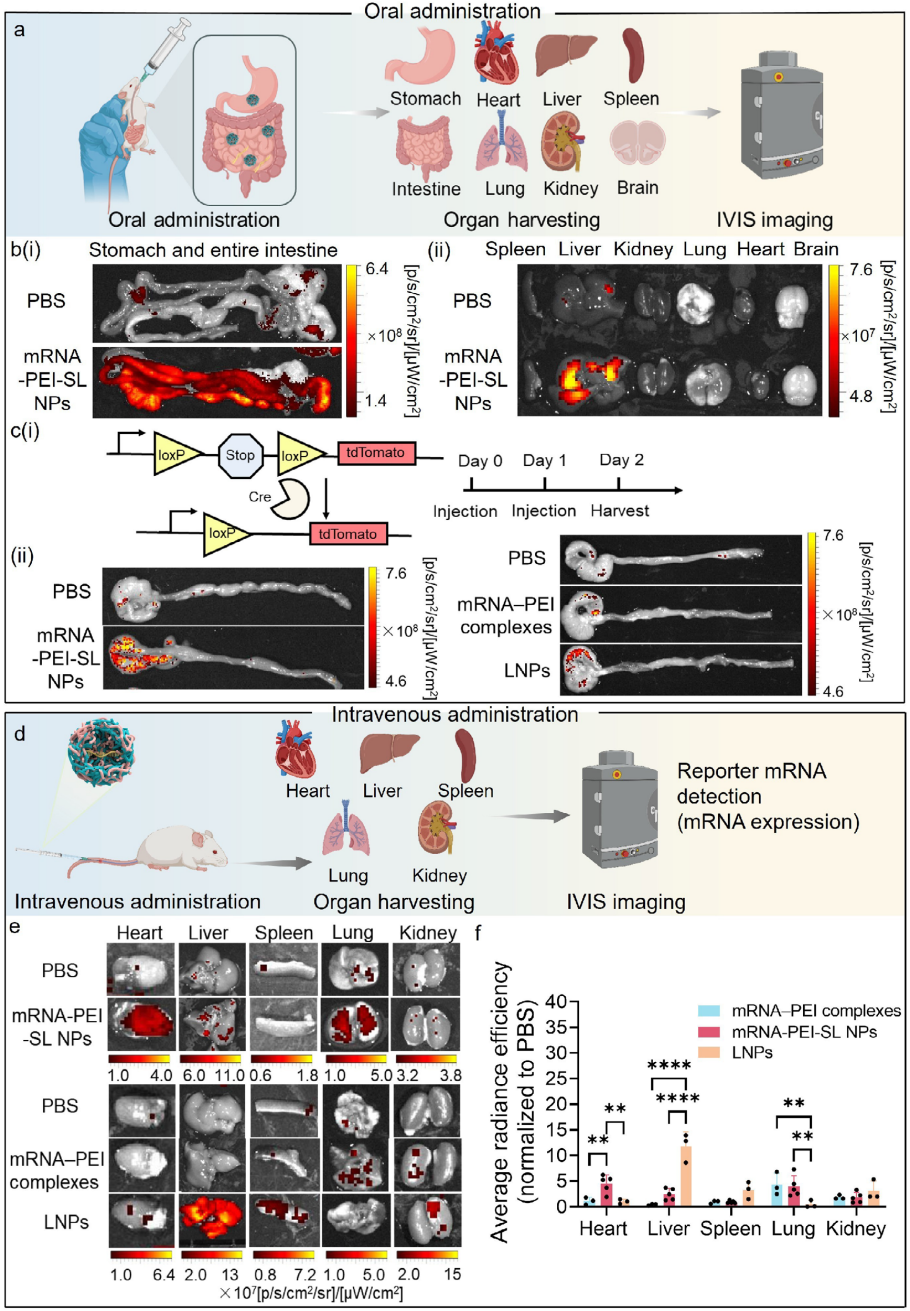
Biodistribution and mRNA transfection in mice using mRNA‐PEI‐SL NPs. (a) Schematic of oral administration of mRNA‐PEI‐SL NPs and subsequent IVIS imaging. (b) Distribution of AF647‐labeled mRNA‐PEI‐SL NPs in the GI tract (i) and major organs after administration of the NPs via oral gavage (ii). (c) Schematic illustration of an Ai14 Cre reporter model (i) and tdTom expression in colon following administration of mRNA–PEI complexes, mRNA‐PEI‐SL NPs, or LNPs via oral gavage (ii). (d) Schematic of intravenous administration of mRNA‐PEI‐SL NPs and subsequent IVIS imaging. (e) mFlame expression and (f) average radiance efficiency of mRNA–PEI complexes, mRNA‐PEI‐SL NPs, and LNPs in different organs. Data are presented as mean values ± SD. Statistical significance was analyzed using one‐way ANOVA with Tukey's multiple comparisons test. ns, not significant; ^*^ *p* < 0.05, ^**^ *p* < 0.01, ^***^ *p* < 0.001, and ^****^ *p* < 0.0001.

Furthermore, SL exhibits a p*K*_a_ of approximately 5.6–7.0, which confers stability in an acidic gastric environment and is dissolved in environments with a pH greater than 7 (e.g., the small intestine and colon) [16]. These properties make SL a promising candidate for protecting mRNA from degradation in the GI tract following oral administration, as it can form a protective layer that shields bioactive cargo from degradation in gastric fluids [25, 26]. Given the previously demonstrated stability of the mRNA‐PEI‐SL NPs in SGF, mucin penetration ability, and effective transfection in vitro, we further examined the potential of the mRNA‐PEI‐SL NPs for oral delivery using mouse models (Figure 3a). For the in vivo oral delivery studies, the mRNA‐PEI‐SL NPs were labeled with Alexa Fluor 647 (AF647) to enable visualization of their biodistribution. As shown in Figure 3b, the mRNA‐PEI‐SL NPs were distributed throughout the GI tract, including the small intestine and colon, 6 h post‐oral administration. Furthermore, the presence of AF647 signal in the liver suggests that a trace amount of NPs may have crossed the epithelial barrier and entered systemic circulation. To assess the ability of the mRNA‐PEI‐SL NPs in mediating oral mRNA delivery in vivo, the genetically engineered tdTomato (tdTom) reporter mice and Cre recombinase (Cre) mRNA were employed. mRNA‐PEI‐SL NPs were injected on two consecutive days to enable cumulative protein expression. In these mice, tdTom expression was initially silenced by a loxP‐flanked stop cassette (Figure 3c). Successful transfection and expression of Cre mRNA would excise the cassette, thereby activating tdTom fluorescence. The tdTom signal observed in the colon implied that the mRNA‐PEI‐SL NPs preserved the activity of mRNA despite exposure to the harsh gastrointestinal environment, whereas mRNA–PEI complexes and SM‐102 lipid NPs (LNPs) led to minimal or no detectable tdTom activation under the same conditions (Figure 3c).

To further explore the in vivo transfection efficacy of the mRNA‐PEI‐SL NPs, a different administration route was examined and mFlame was encapsulated within the mRNA‐PEI‐SL NPs. mFlame is an engineered mRNA encoding brighter red fluorescence proteins (see Experimental Section). The mFlame‐encapsulated mRNA‐PEI‐SL NPs were injected intravenously into C57BL/6J mice. Major organs, including the liver, spleen, kidney, lung, and heart, were harvested after 24 h and imaged using an In Vivo Imaging System (IVIS) (Figure 3d). Unlike SM‐102 LNPs that typically enabled pronounced mRNA expression in the liver, mRNA‐PEI‐SL NPs mediated mRNA transfection primarily in the heart and lung (Figure 3e,f). Accordingly, the fluorescence intensity in these organs were 4.3‐ and 8.5‐fold higher, respectively, compared to those in the SM‐102 LNP‐treated mice. This variation in mRNA expression tropism is likely attributed to the protein corona formed on NPs, which is known to influence in vivo biodistribution and organ‐specific accumulation [27]. Although additional studies are required to determine the exact mechanism, this accumulation suggests potential of the SL NP platform for heart‐ and lung‐tropic mRNA delivery.

## 3 Conclusion

We developed a versatile, modular SL‐enabled NP platform for mRNA delivery, including oral delivery. The versatile binding affinity of SL, combined with its neutral‐to‐negative charge, facilitates the assembly of a compact structure and shields the charge of the cationic molecules assembled within the NPs. The SL‐based NPs exhibited high mRNA encapsulation efficiency (>90%) and demonstrated transfection efficacy both in vitro and in vivo. Notably, this platform protected mRNA from degradation in the harsh gastrointestinal environment, enabling transfection in the colon after oral administration. This work presents a promising alternative mRNA delivery and gene editing platform and provides a viable strategy for the oral administration of nucleic acid therapeutics to potentially treat intestinal and colonic diseases.

## Conflicts of Interest

F.C. is a shareholder of Messenger Bio Pty Ltd. The remaining authors declare no conflicts of interest.

## Supporting information




**Supporting File**: adhm71156‐sup‐0001‐SuppMat.pdf.

## Data Availability

The data that support the findings of this study are available from the corresponding author upon reasonable request.

## References

[1] M. Metkar , C. S. Pepin , and M. J. Moore , “Tailor Made: The Art of Therapeutic mRNA Design,” Nature Reviews Drug Discovery 23 (2024): 67–83, 10.1038/s41573-023-00827-x. 38030688

[2] X. Huang , N. Kong , X. Zhang , Y. Cao , R. Langer , and W. Tao , “The Landscape of mRNA Nanomedicine,” Nature Medicine 28 (2022): 2273–2287, 10.1038/s41591-022-02061-1. 36357682

[3] X. Hou , T. Zaks , R. Langer , and Y. Dong , “Lipid Nanoparticles for mRNA Delivery,” Nature Reviews Materials 6 (2021): 1078–1094, 10.1038/s41578-021-00358-0. 34394960 PMC8353930

[4] Q. Shuai , F. Zhu , M. Zhao , and Y. Yan , “mRNA Delivery via Non‐Viral Carriers for Biomedical Applications,” International Journal of Pharmaceutics 607 (2021): 121020, 10.1016/j.ijpharm.2021.121020. 34416327

[5] B. Zhao , X. Zhang , M. S. Bickle , S. Fu , Q. Li , and F. Zhang , “Development of Polypeptide‐Based Materials Toward Messenger RNA Delivery,” Nanoscale 16 (2024): 2250–2264, 10.1039/D3NR05635J. 38213302

[6] F. Richter , K. Leer , L. Martin , et al., “The Impact of Anionic Polymers on Gene Delivery: How Composition and Assembly Help Evading the Toxicity‐Efficiency Dilemma,” Journal of Nanobiotechnology 19 (2021): 1–15, 10.1186/s12951-021-00994-2. 34579715 PMC8477462

[7] Y. J. Choi , S. J. Kang , Y. J. Kim , Y.‐B. Lim , and H. W. Chung , “Comparative Studies on the Genotoxicity and Cytotoxicity of Polymeric Gene Carriers Polyethylenimine (PEI) and Polyamidoamine (PAMAM) Dendrimer in Jurkat T‐Cells,” Drug and Chemical Toxicology 33 (2010): 357–366, 10.3109/01480540903493507. 20550436

[8] C. Meng , Z. Chen , G. Li , T. Welte , and H. Shen , “Nanoplatforms for mRNA Therapeutics,” Advanced Therapeutics 4 (2021): 2000099, 10.1002/adtp.202000099.

[9] J. Ren , Y. Cao , L. Li , et al., “Self‐Assembled Polymeric Micelle as a Novel mRNA Delivery Carrier,” Journal of Controlled Release 338 (2021): 537–547, 10.1016/j.jconrel.2021.08.061. 34481924 PMC8411660

[10] W. Godbey , K. K. Wu , and A. G. Mikos , “Size Matters: Molecular Weight Affects the Efficiency of Poly(ethylenimine) as a Gene Delivery Vehicle,” Journal of Biomedical Materials Research 45 (1999): 268–275, 10.1002/(SICI)1097-4636(19990605)45:3<268::AID-JBM15>3.0.CO;2-Q. 10397985

[11] P. Y. Teo , C. Yang , J. L. Hedrick , et al., “Hydrophobic Modification of Low Molecular Weight Polyethylenimine for Improved Gene Transfection,” *Biomaterials* 34 (2013): 7971–7979. 10.1016/j.biomaterials.2013.07.005 23880339

[12] P. Huang , H. Deng , Y. Zhou , and X. Chen , “The Roles of Polymers in mRNA Delivery,” Matter 5 (2022): 1670–1699.

[13] M. Li , M. Zhao , Y. Fu , et al., “Enhanced Intranasal Delivery of mRNA Vaccine by Overcoming the Nasal Epithelial Barrier via Intra‐and Paracellular Pathways,” Journal of Controlled Release 228 (2016): 9–19, 10.1016/j.jconrel.2016.02.043. 26941035

[14] Y. Farag and C. S. Leopold , “Development of Shellac‐Coated Sustained Release Pellet Formulations,” European Journal of Pharmaceutical Sciences 42 (2011): 400–405, 10.1016/j.ejps.2011.01.006. 21251975

[15] J. Wang , X. Wang , B. Liu , J. Xiao , and Z. Fang , “Shellac‐based films/coatings: Progress, applications and future trends in the field of food packaging,” Food Chemistry 467 (2025): 142326, 10.1016/j.foodchem.2024.142326. 39644663

[16] Y. Yuan , N. He , L. Dong , et al., “Multiscale Shellac‐Based Delivery Systems: From Macro‐to Nanoscale,” ACS Nano 15 (2021): 18794–18821, 10.1021/acsnano.1c07121. 34806863

[17] Q. Luo , K. Li , J. Xu , et al., “Novel Biobased Sodium Shellac for Wrapping Disperse Multiscale Emulsion Particles,” Journal of Agricultural and Food Chemistry 64 (2016): 9374–9380, 10.1021/acs.jafc.6b04417. 27960293

[18] Y. Chen , Z. Zhu , K. Shi , et al., “Shellac‐Based Materials: Structures, Properties, and Applications,” International Journal of Biological Macromolecules 279 (2024): 135102, 10.1016/j.ijbiomac.2024.135102. 39197605

[19] S. Hu , Z. Yang , S. Wang , et al., “Zwitterionic Polydopamine Modified Nanoparticles as an Efficient Nanoplatform to Overcome both the Mucus and Epithelial Barriers,” Chemical Engineering Journal 428 (2022): 132107, 10.1016/j.cej.2021.132107.

[20] Y. Xiao , Z. Tang , X. Huang , et al., “Glucose‐Responsive Oral Insulin Delivery Platform for One Treatment a Day in Diabetes,” *Matter* 4 (2021): 3269–3285.

[21] Q. Yang , H. Dai , B. Wang , et al., “Nanoplastics Shape Adaptive Anticancer Immunity in the Colon in Mice,” Nano Letters 23 (2023): 3516–3523, 10.1021/acs.nanolett.3c00644. 37043775

[22] F. Hausig‐Punke , F. Richter , M. Hoernke , J. C. Brendel , and A. Traeger , “Tracking the Endosomal Escape: A Closer Look at Calcein and Related Reporters,” Macromolecular Bioscience 22 (2022): 2200167, 10.1002/mabi.202200167. 35933579

[23] J. I. Solomun , G. Cinar , P. Mapfumo , et al., “Solely Aqueous Formulation of Hydrophobic Cationic Polymers for Efficient Gene Delivery,” International Journal of Pharmaceutics 593 (2021): 120080, 10.1016/j.ijpharm.2020.120080. 33246046

[24] M. Faria , M. Björnmalm , K. J. Thurecht , et al., “Minimum Information Reporting in Bio–Nano Experimental Literature,” Nature Nanotechnology 13 (2018): 777–785. 10.1038/s41565-018-0246-4 PMC6150419 30190620

[25] C. Zhang , Z. Chen , Y. He , et al., “Oral Colon‐Targeting Core–Shell Microparticles Loading Curcumin for Enhanced Ulcerative Colitis Alleviating Efficacy,” Chinese Medicine 16 (2021): 1–14. 34551815 10.1186/s13020-021-00449-8 PMC8456585

[26] K. Wang , H.‐F. Wen , D.‐G. Yu , Y. Yang , and D.‐F. Zhang , “Electrosprayed Hydrophilic Nanocomposites Coated With Shellac for Colon‐Specific Delayed Drug Delivery,” Materials & Design 143 (2018): 248–255, 10.1016/j.matdes.2018.02.016.

[27] S. A. Dilliard , Q. Cheng , and D. J. Siegwart , “On the Mechanism of Tissue‐Specific mRNA Delivery by Selective Organ Targeting Nanoparticles,” Proceedings of the National Academy of Sciences 118 (2021): 2109256118, 10.1073/pnas.2109256118. PMC8719871 34933999

