# Supplementary material for: Shellac‐Mediated Assembly of Nanoparticles for mRNA Delivery
Source: Adv Healthc Mater. 2026 Apr 21;15(23):e05918. doi: 10.1002/adhm.202505918 (PMC13280163; doi:10.1002/adhm.202505918)
Supplement: Supplementary file 1 — Supporting File: adhm71156‐sup‐0001‐SuppMat.pdf. [file ADHM-15-0-s001.pdf]

Supporting Information

Shellac-Mediated Assembly of Nanoparticles for mRNA Delivery

*Meizhang Lu, Jingqu Chen, Zhixing Lin, Wanjun Xu, Tianzheng Wang, Shiyao Li, Die Yang, and Frank Caruso**

M. Lu, Dr. J. Chen, Dr. Z. Lin, Dr. W. Xu, T. Wang, Dr. S. Li, Dr. D. Yang, Prof. F. Caruso

Department of Chemical Engineering, The University of Melbourne, Parkville, Victoria 3010, Australia

E-mail: fcaruso@unimelb.edu.au

Experimental Section

Materials. Polyethyleneimine (PEI; 800 Da and 25 kDa), chitosan (low molecular weight, 50–190 kDa), poly-L-lysine (15–30 kDa), urea, Tween 20, and sodium chloride were purchased from Sigma-Aldrich (USA). Wax-free shellac (SL) was purchased from AA Blocks. Alexa Fluor 647 NHS ester, Dulbecco's modified Eagle medium (DMEM), fetal bovine serum (FBS), LysoTracker deep green, 2,3-bis[2-methoxy-4-nitro-5-sulfophenyl]-2*H*-tetrazolium-5-carboxyanilide inner salt (XTT), trihydrochloride (Hoechst 33342, 10 mg mL^{-1}), and Dulbecco's phosphate-buffered saline (DPBS) were obtained from Life Technologies. Firefly luciferase (Luc), mCherry, mFlame, and cyanine 5 (Cy5)-labeled mRNA were synthesized by Messenger Bio Pty Ltd. (Australia). 1-Octylnonyl ester (SM-102) was purchased from APExBIO (TX, USA). 1,2-Distearoyl-*sn*-glycero-3-phosphocholine (DSPC), cholesterol, and 1,2-dimyristoyl-*rac*-glycero-3-methoxypolyethylene glycol-2000 (DMG-PEG 2000) were purchased from Avanti Polar Lipids (AL, USA). Tumor necrosis factor-α (TNF-α) enzyme-linked immunosorbent assay kit and Quant-iT RiboGreen RNA assay kit were purchased from Thermo Fisher Scientific. Bright-Glo luciferase assay system and VivoGlo luciferin were obtained from Promega. Caco-2 cells, HEK293T cells, and RAW 264.7 cells were purchased from the American Type Culture Collection.

Assembly of mRNA-Loaded Cationic Molecule (CM)-Shellac Nanoparticles (mRNA-CM-SL NPs). To prepare mRNA-CM-SL NPs, PEI (10 μL, 1 mg mL^{-1} in RNase-free water), SM-102 (14 μL, 1 mM in ethanol), chitosan (10 μL, 1 mg mL^{-1} in acetic acid), or PLys (10 μL, 1 mg mL^{-1} in RNase-free water) was first added to a 1.7 mL Eppendorf tube containing RNase-free water (289 μL) and mRNA (1 μL, 1 mg mL^{-1} in RNase-free water) and then incubated for 10 min. SL (2 μL, 5 mg mL^{-1} in ethanol) was added in another tube containing ethanol (98 μL). An aliquot (100 μL) of the SL solution was then rapidly added to the aqueous solution and incubated for 10 min.

Formation of Lipid NPs (LNPs). LNPs were prepared on a NanoAssemblr Ignite. mRNA was dissolved in 10 mM citrate buffer to form the aqueous phase. The lipid phase consisted of SM-102, DSPC, cholesterol, and DMG-PEG 2000 at a molar ratio of 50:10:38.5:1.5. The two phases were mixed at an aqueous-to-lipid ratio of 3:1 at a flow rate of 12 mL min^{-1}, and then dialyzed overnight in DPBS using a 6 kDa cutoff dialysis tube.

Characterization. Transmission electron microscopy was performed on an FEI Tecnai TF20 instrument (USA) at an operating voltage of 200 kV. Size and ξ-potential were measured via dynamic light scattering (DLS) on a Malvern Zetasizer Nano ZS instrument (Malvern Instrument, UK). UV–Visible absorption spectra were recorded on a Specord 250 Plus spectrophotometer (Analytik Jena AG). Fourier transform infrared spectroscopy analysis was performed on a Tensor II FTIR spectrometer (Bruker Optics, USA). Confocal laser scanning microscopy (CLSM) images were taken with a Nikon A1R+ laser scanning confocal microscope (Nikon Corporation, Japan).

Encapsulation Efficiency. The mRNA-PEI-SL NPs with 1 µg mRNA were centrifuged at 8000 *g* for 5 min. The supernatant was withdrawn and quantified using the Quant-iT RiboGreen RNA assay. The fluorescence of the collected supernatant was measured on an Infinite M200 microplate reader (Tecan, Switzerland) at an excitation of 485 nm and emission of 525 nm. The encapsulation efficiency (%) of mRNA was calculated using the following equation:

$$\text{Encapsulation efficiency (\%)} = \frac{m_{\text{total mRNA}} - m_{\text{free mRNA}}}{m_{\text{total mRNA}}} \times 100\%$$

where $m_{\text{total mRNA}}$ is the total amount of mRNA initially added and $m_{\text{free mRNA}}$ is the amount of unencapsulated mRNA detected in the supernatant.

In Vitro Release Studies. The mRNA-PEI-SL NPs were incubated in DMEM or DPBS at 37 °C, separately. At the designated time points, the NP suspension was centrifuged at 8000 *g* for 5 min. The supernatant was removed and replaced with an equal volume of fresh medium. The fluorescence of the collected supernatant was measured using the Quant-iT RiboGreen RNA assay.

Disassembly Assay. To determine the possible driving forces for the assembly of the mRNA-PEI-SL NPs, the NPs were incubated in urea, Tween 20, or NaCl for the desired time. Changes in NP size were measured on a Zetasizer. Data are shown as the mean ± standard deviation (SD) of three independent measurements.

Stability Assay. To evaluate the stability of the mRNA-PEI-SL NPs under different conditions, the NPs were dispersed in RNase-free water or DMEM+10% FBS at 37 °C for the desired time. Changes in the NP size were measured on a Zetasizer. Data are shown as the mean ± SD of three independent measurements.

Enzymatic and pH Stability. To evaluate the enzymatic and pH stability of the mRNA-PEI-SL NPs, pepsin and trypsin were used as model proteases. Pepsin was added to pH 1.2 buffers to obtain simulated gastric fluid (SGF) solutions, whereas trypsin was added to pH 6.8 buffers to obtain simulated intestinal fluid (SIF) solutions. mRNA-PEI-SL NPs were dispersed in SGF or SIF for the desired time. Changes in the NP size were measured on a Zetasizer. Data are shown as the mean ± SD of three independent measurements.

Mucin Penetration. To evaluate the mucin penetration ability of the mRNA-PEI-SL NPs, agarose powder was dissolved to achieve a final concentration of 3 mg mL^{-1}. Then, an aliquot (1 mL) of the agarose gel solution was added to a vial. After the agarose solution had hardened, an aliquot (1 mL) of a 10 mg mL^{-1} mucin solution was added to the agarose gel. Then, an aliquot (200 µL) of 10 mg mL^{-1} AF647-labeled mRNA-PEI-SL NPs was added to the mucus layer and incubated at 37 °C. At the designated time points, the mucus layer was removed, and the

agarose gel was washed three times, melted, and the fluorescence was measured using the Infinite M200 microplate reader (Tecan, Switzerland).

In addition, mRNA-PEI-SL NPs were incubated with a mucin solution for 1 h, and the size of the mucin-coated mRNA-PEI-SL NPs before and after incubation was measured using a Zetasizer.

Cell Culture. HEK293T and RAW264.7 cells were cultured in complete DMEM medium with 10% FBS and 1% streptomycin/penicillin. Caco-2 cells were cultured in supplemented RPMI-1640. Cells were grown at 37 °C, 5% CO_2, and 95% humidity.

Cell Viability by XTT Assay. HEK293T, Caco2, and RAW264.7 cells were seeded at a density of 1 × 10^4 cells per well on a 96-well plate at 37 °C overnight. Then, the culture media was discarded and replaced with fresh media, which contained NPs at different concentrations, for 24 h. After the treatment, the media was replaced with fresh media containing activated XTT, and cells were further incubated for 3 h. Finally, cells were screened on an Infinite M200 microplate reader (Tecan, Switzerland); absorbance readings were measured at 475 nm, and a reference wavelength of 675 nm was used. Cell viability was expressed as a percentage by normalizing the absorbance to that of untreated cells. All experiments were performed in quadruplicate, and data are presented as the mean ± SD.

Cytokine Assays. Plasma was collected from mice treated with DPBS, LNP, mRNA–PEI complexes, or mRNA-PEI-SL NPs through intravenous injection by cardiac puncture and then centrifuged at 900 *g* for 15 min. The TNF-α levels were determined using an enzyme-linked immunosorbent assay (ELISA) kit according to the manufacturer’s instructions.

Cell Association of mRNA-PEI-SL NPs. HEK293T cells were seeded at a density of 5 × 10^4 cells per well on a 24-well plate at 37 °C overnight. Then, the culture media was removed and replaced with fresh media containing AF647-labeled mRNA-PEI-SL NPs at a dose of 1 μg of Luc-mRNA for 24 h. After incubation, the cells were gently washed three times with DPBS, dissociated using trypsin solution, and analyzed on a BD Accuri C6 Plus. The degree of cell association of the particles was evaluated by using the percentage of cells that exhibited a stronger fluorescence intensity than the control, untreated cells.

Mechanism of Internalization of mRNA-PEI-SL NPs. HEK293T cells were seeded at a density of 5 × 10^4 cells per well on a 24-well plate at 37 °C overnight. Then, the culture media was removed and endocytosis inhibitors (pitstop2, EIPA, filipin from *Streptomyces filipinensis*, and cytochalasin D) were added to the cells to achieve final concentrations of 5, 15, 12 μg mL^{-1}, and 25 μM, respectively. After 15 min incubation with the endocytosis inhibitors, AF647-labeled mRNA-PEI-SL NPs at a dose of 1 μg of Luc-mRNA were added to cells for 24 h. After incubation,

the cells were gently washed three times with DPBS, dissociated using trypsin solution, and analyzed on a BD LSRFortessa instrument.

In Vitro Transfection. HEK293T cells were seeded at a density of 5 × 10^4 cells per well on a 24-well plate at 37 °C overnight. Then, the culture media was removed and replaced with fresh media, and then treated with mRNA, mRNA–PEI complexes, mRNA-PEI-SL NPs, or MessengerMax-transfected mRNA for 24 h. The concentration of mRNA in each formulation was 1 μg mL^{-1}. After incubation, the cells were gently washed three times with DPBS, dissociated using trypsin solution, and analyzed on a BD Accuri C6 Plus. The degree of cell association of the particles was evaluated by using the percentage of cells that exhibited a stronger fluorescence intensity than the control, untreated cells.

For CLSM imaging following mRNA transfection in simulated gastrointestinal fluids, Caco-2 cells were seeded on 8-well Lab-Tek chamber slides. mRNA-PEI-SL NPs were first incubated with SGF for 30 min, and then in SIF for 30 min, and finally delivered to the cells for 24 h.

Endosomal Escape. HEK293T cells were seeded on an 8-well Lab-Tek-Chamber slide at a density of 4 × 10^4 cells per well for 24 h. Then, mRNA-PEI-SL NPs were added to cells and incubated for 12 h. After incubation, the cells were gently washed with DPBS and stained with Lysotracker Green for 1 h and then gently washed with DPBS. Cells were finally stained with Hoechst 33342 (2 μg mL^{-1}) for 10 min to stain the cell nuclei. The solution was aspirated and replaced with DPBS. Live cell imaging was performed via CLSM using a Plan Apo λ 40× 1.4 NA water immersion objective, and lasers at 405, 488, 561, and 640 nm, along with 450/50, 525/50, 595/50, and 700/75 nm bandpass emission filters. The images were processed by Fiji software.

Ethics

This work was conducted in accordance with the Australian code for the care and use of animals for scientific purposes, and experiments were approved by The University of Melbourne Animal Ethics Committee (Ethics no. 27608).

In Vivo Transfection Via Intravenous Administration. Mice were sourced from the Bioresources Facility of the Peter Doherty Institute (Melbourne, Australia) and housed under a 12-hour light/dark cycle with ad libitum access to food and water.

Briefly, mRNA–PEI complexes, mRNA-PEI-SL NPs, and LNPs were injected into C57BL/6J mice (female, 8–10 weeks old) at a dosage of 5 μg of mRNA per mouse (~0.25 mg kg^{-1}) via the lateral tail vein, while the same volume of DPBS was injected as a negative control. The mice were euthanized by CO_2 asphyxiation at designated time points. Major organs, including the heart, liver, spleen, lungs, and kidneys, were harvested post-injection, followed by imaging on an In Vivo Imaging System (PerkinElmer, USA). Spectral unmixing was conducted using DPBS-

treated mice to unmix the auto-tissue fluorescence. Five biologically independent mice were included in each group.

Biodistribution of mRNA-PEI-SL NPs Administered Via Oral Gavage in Mice. AF647-labeled-mRNA-PEI-SL NPs were administered into mice via oral gavage at a dosage of 10 µg of mRNA-Luc per mouse. The mice were euthanized by CO_2 asphyxiation 6 h post-injection, and the major organs were collected. Two biologically independent mice were included in each group.

In Vivo Transfection by mRNA-PEI-SL NPs Administered Via Oral Gavage in Mice. mRNA–PEI complexes, mRNA-PEI-SL NPs, and LNPs were injected into B6.Cg-Gt(ROSA)26Sor$^{tm14(CAG\text{-}tdTomato)Hze/J}$ mice (Common name: Ai14; mixed gender, 8–10 weeks old) at a dosage of 10 µg of mRNA per mouse (~0.5 mg kg^{-1}) via oral gavage at Day 0 and Day 1, while the same volume of DPBS was injected as a negative control. On Day 2, mice were euthanized, and major organs were collected. Three biologically independent mice were included in each group.

Minimum Information Reporting in Bio–Nano Experimental Literature (MIRIBEL). The studies conducted herein, including material characterization, biological characterization, and experimental details, conform to the MIRIBEL reporting standard for bio–nano research,[1] and we include a companion checklist of these components herein.

Statistical Analysis. Statistical analysis was performed using GraphPad Prism v.9.0 software. The data were presented as mean ± SD. Statistical significance was assessed by one-way and two-way analysis of variance (ANOVA) with Tukey's multiple comparisons test. The significance was defined as follows: ns, not significant; $^{*}P < 0.05$, $^{**}P < 0.01$, $^{***}P < 0.001$, and $^{****}P < 0.0001$.

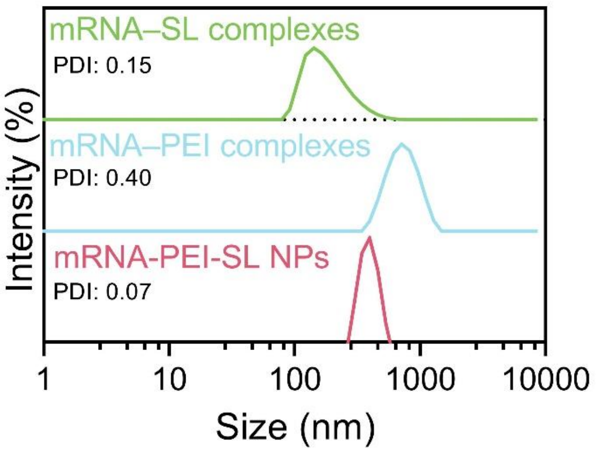

Figure S1. Size distribution of mRNA–SL complexes, mRNA–PEI complexes, and mRNA-PEI-SL NPs, as determined by DLS.

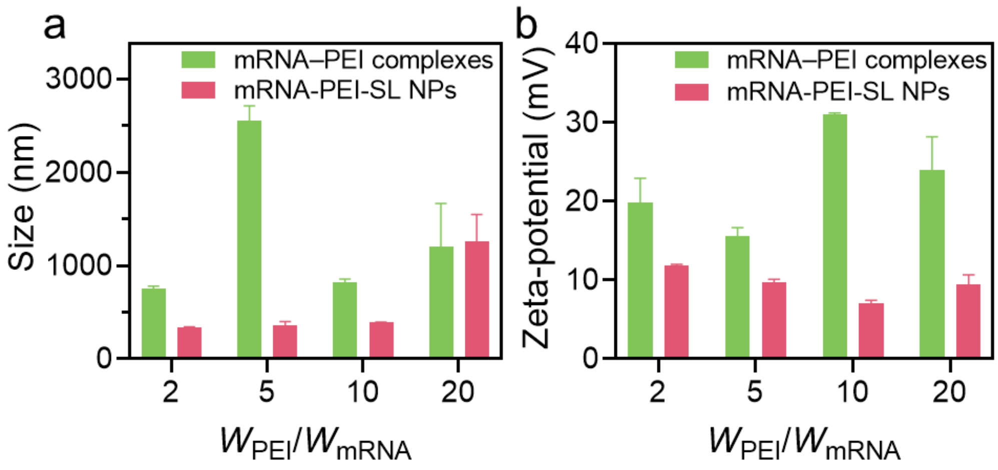

Figure S2. (a) Size and (b) zeta-potential of the mRNA–PEI complexes and mRNA-PEI-SL NPs in water, as determined by DLS, as a function of PEI-to-mRNA mass ratio (W_{PEI}/W_{mRNA}). Data are presented as the mean ± SD, n = 3.

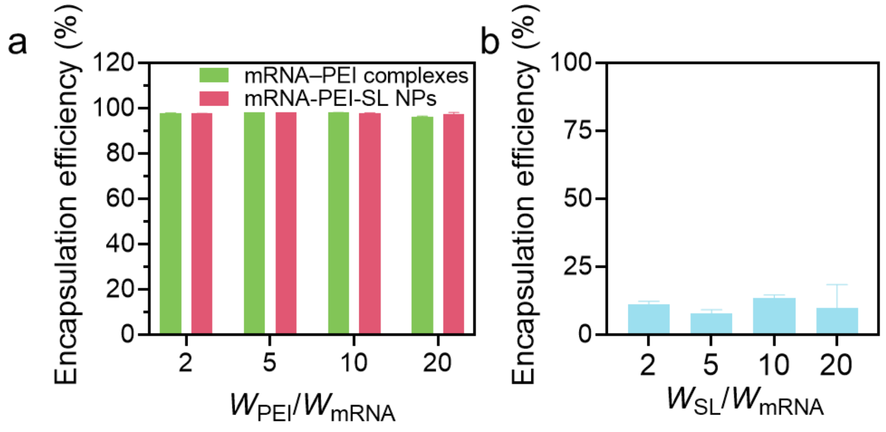

Figure S3. mRNA encapsulation efficiency of (a) mRNA–PEI complexes and mRNA-PEI-SL NPs, and (b) mRNA–SL complexes as a function PEI-to-mRNA and SL-to-mRNA mass ratios, respectively.

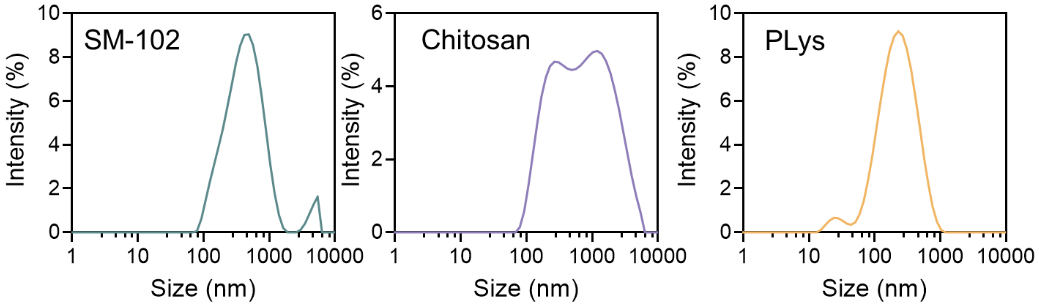

Figure S4. Size distribution of mRNA–CM complexes assembled from different cationic molecules (CMs; SM-102, chitosan, or PLys), as determined by DLS.

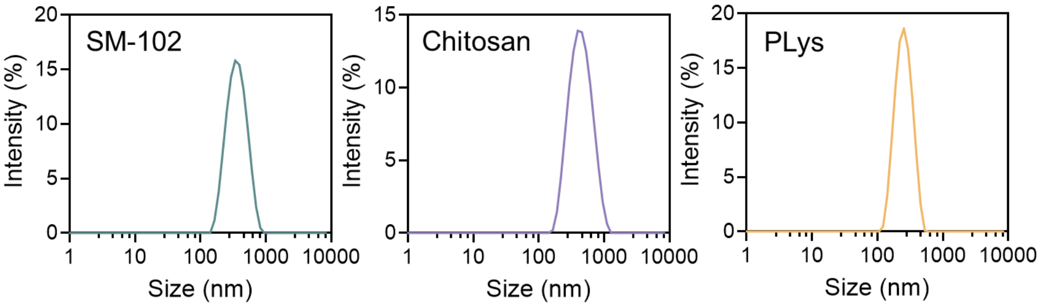

Figure S5. Size distribution of mRNA-CM-SL NPs assembled from different cationic molecules (CMs; SM-102, chitosan, or PLys), as determined by DLS.

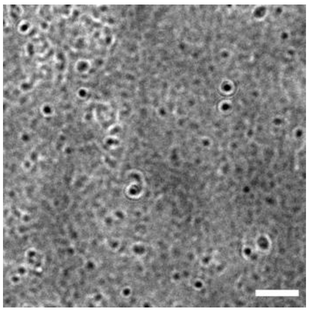

Figure S6. Representative transmission electron microscopy image of mRNA-PLys-SL NPs; scale bar is 200 nm.

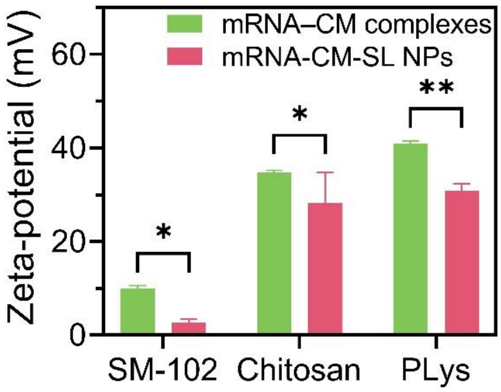

Figure S7. Zeta-potential of mRNA–CM complexes and mRNA-CM-SL NPs assembled from different cationic molecules (CMs; SM-102, chitosan, or PLys), as determined by DLS. Data are presented as the mean ± SD, n = 3. One-way ANOVA with Tukey's multiple comparisons test was performed: *P < 0.05.

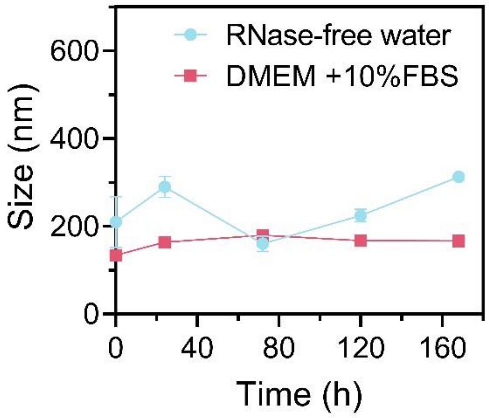

Figure S8. Stability of mRNA-PEI-SL NPs upon incubation in different milieu at 37 °C. Data are presented as the mean ± SD, n = 3.

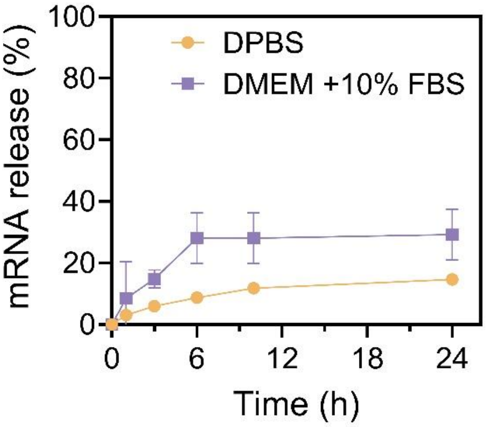

Figure S9. Release of mRNA from mRNA–PEI complexes upon incubation in different milieu at 37 °C. Data are presented as the mean ± SD, *n* = 3.

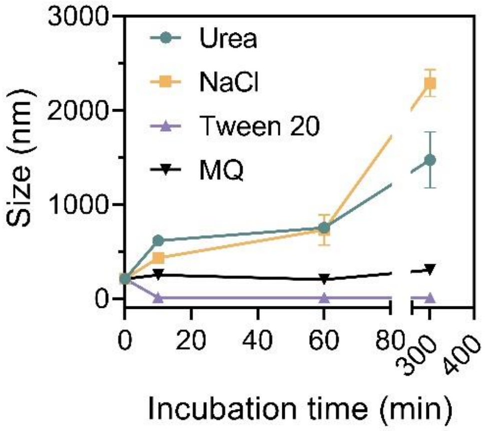

Figure S10. Stability of mRNA-PEI-SL NPs upon incubation in different buffers. Data are presented as the mean ± SD, n = 3. MQ, milli-Q water.

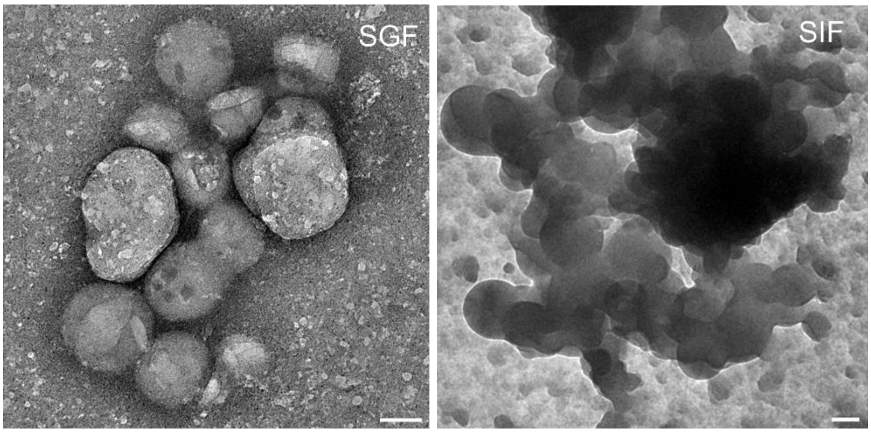

Figure S11. Transmission electron microscopy images of mRNA-PEI-SL NPs upon incubation in SGF and SIF for 24 h. Scale bars are 100 nm.

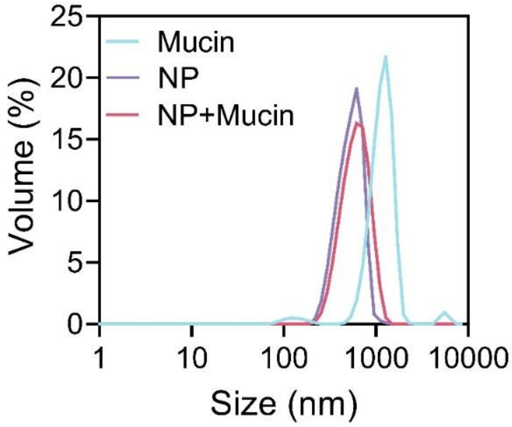

Figure S12. Stability of mRNA-PEI-SL NPs upon incubation in mucin for 24 h.

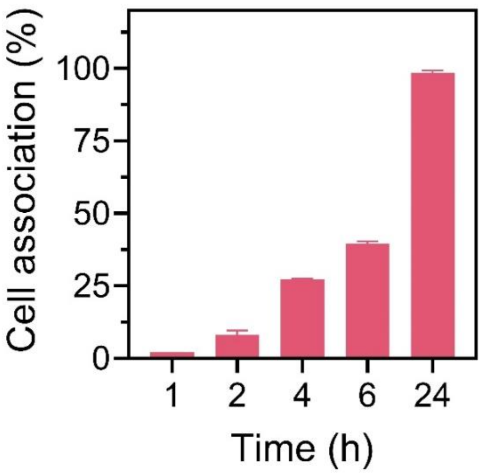

Figure S13. Time-dependent association of the mRNA-PEI-SL NPs with HEK293T cells. The mRNA-PEI-SL NPs were assembled with mRNA, AF647-labeled PEI, and SL at a mass ratio of 1:10:10. Data are presented as the mean ± SD, $n = 3$.

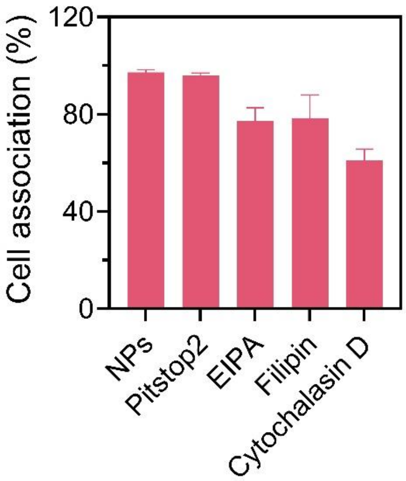

Figure S14. Evaluation of the cellular uptake pathways of mRNA-PEI-SL NPs using endocytic inhibitors. The mRNA-PEI-SL NPs were assembled with mRNA, AF647-labeled PEI, and SL at a mass ratio of 1:10:10. Data are presented as the mean ± SD, n = 3.

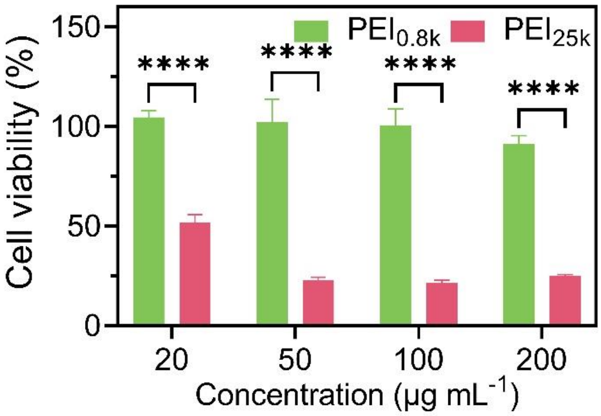

Figure S15. Cytotoxicity of $PEI_{0.8k}$ and PEI_{25k} toward HEK293T cells. Viability of HEK293T cells after incubation with PEI at different concentrations. Data are presented as the mean ± SD (n = 5). Two-way ANOVA was performed: ***$P < 0.001$.

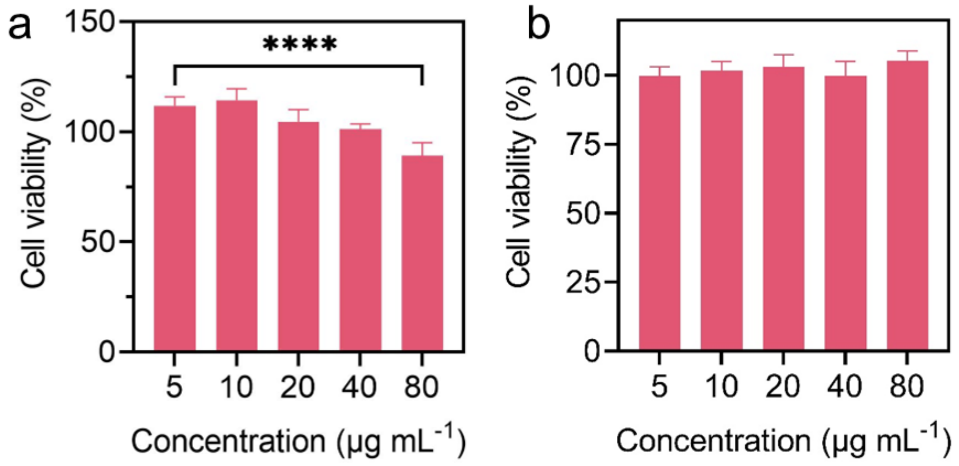

Figure S16. Cytotoxicity of mRNA-PEI-SL NPs toward (a) Caco-2 cells and (b) RAW264.7 cells. Viability of Caco-2 and RAW264.7 cells after incubation with mRNA-PEI-SL NPs at different NP concentrations. Data are presented as the mean ± SD (n = 5). The mRNA-PEI-SL NPs were assembled with mRNA, PEI, and SL at a mass ratio of 1:10:10. One-way ANOVA was performed: ****$P < 0.0001$.

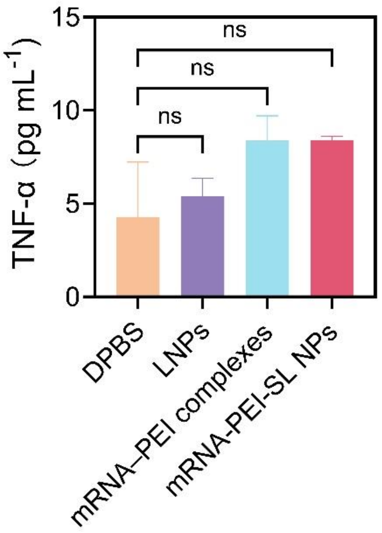

Figure S17. ELISA measurements of the release of TNF-α in plasma collected from mice treated with DPBS, LNPs, mRNA–PEI complexes, or mRNA-PEI-SL NPs through intravenous injection. Data are presented as mean ± SD. Statistical significance was analyzed using one-way ANOVA with Tukey's multiple comparisons test. The significance was defined as follows: ns, not significant; **P* < 0.05, ***P* < 0.01, ****P* < 0.001, and *****P* < 0.0001.

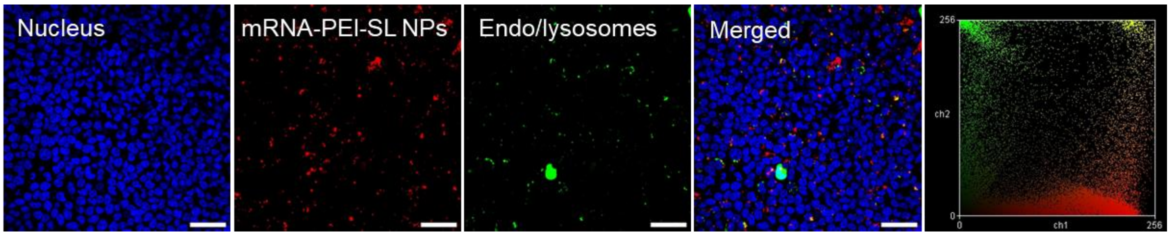

Figure S18. Endosomal escape of mRNA-PEI-SL NPs. CLSM images of HEK293T cells incubated with mRNA-PEI-SL NPs overnight. Red, mRNA-PEI-SL NPs assembled with Cy5-labeled mRNA; green, endosomes and lysosomes; blue, nuclei. Scale bars are 50 μm. The color scatter plot shows the correlation between green and red fluorescence signals.

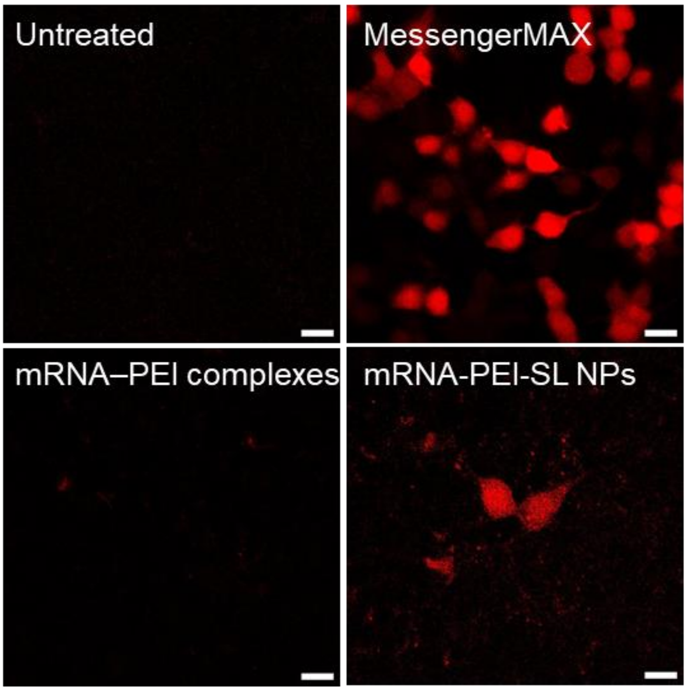

Figure S19. CLSM images of HEK293T cells after 24 h treatment without (untreated group) or with MessengerMAX, mRNA–PEI complexes, or mRNA-PEI-SL NPs. Scale bars are 10 μm. The mRNA-PEI-SL NPs were assembled with mRNA, PEI, and SL at a mass ratio of 1:10:10.

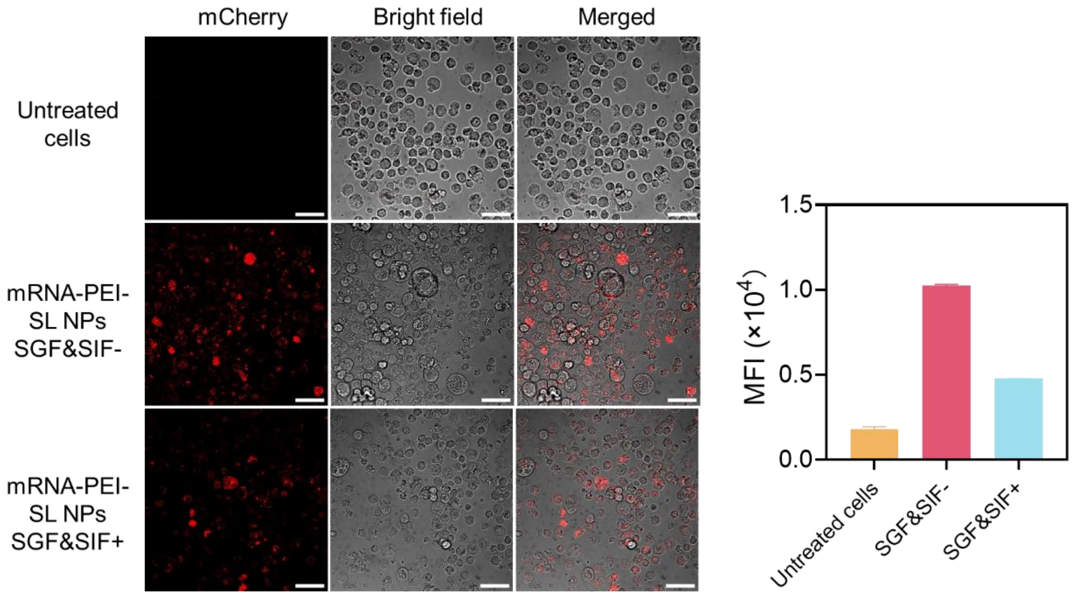

Figure S20. CLSM images and mean fluorescence intensity (MFI) data showing the transfection of mRNA-PEI-SL NPs with or without pre-treatment of SGF and SIF in Caco-2 cells after 24 h. Scale bars are 50 μm. The mRNA-PEI-SL NPs were assembled with mRNA, PEI, and SL at a mass ratio of 1:10:10.

Checklist

Minimum Information Reporting in Bio–Nano Experimental Literature

The MIRIBEL guidelines were introduced here: https://doi.org/10.1038/s41565-018-0246-4

The development of these guidelines was led by the ARC Centre of Excellence in Convergent Bio-Nano Science and Technology: https://www.cbns.org.au/. Any updates or revisions to this document will be made available here: http://doi.org/10.17605/OSF.IO/SMVTF. This document is made available under a CC-BY 4.0 license: https://creativecommons.org/licenses/by/4.0/.

The MIRIBEL guidelines were developed to facilitate reporting and dissemination of research in bio–nano science. Their development was inspired by various similar efforts:

- MIAME (microarray experiments): *Nat. Genet.* **29** (2001), 365; http://doi.org/10.1038/ng1201-365
- MIRIAM (biochemical models): *Nat. Biotechnol.* **23** (2005) 1509; http://doi.org/10.1038/nbt1156
- MIBBI (biology/biomedicine): *Nat. Biotechnol.* **26** (2008) 889; http://doi.org/10.1038/nbt.1411
- MIGS (genome sequencing): *Nat. Biotechnol.* **26** (2008) 541; http://doi.org/10.1038/nbt1360
- MIQE (quantitative PCR): *Clin. Chem.* **55** (2009) 611; http://doi.org/10.1373/clinchem.2008.112797
- ARRIVE (animal research): *PLOS Biol.* **8** (2010) e1000412; http://doi.org/10.1371/journal.pbio.1000412
- *Nature*'s reporting standards:
  - Life science: https://www.nature.com/authors/policies/reporting.pdf; e.g., *Nat. Nanotechnol.* **9** (2014) 949; http://doi.org/10.1038/nnano.2014.287
  - Solar cells: https://www.nature.com/authors/policies/solarchecklist.pdf; e.g., *Nat. Photonics* **9** (2015) 703; http://doi.org/10.1038/nphoton.2015.233
  - Lasers: https://www.nature.com/authors/policies/laserchecklist.pdf; e.g., *Nat. Photonics* **11** (2017) 139; http://doi.org/10.1038/nphoton.2017.28
- The "TOP guidelines": e.g., *Science* **352** (2016) 1147; http://doi.org/10.1126/science.aag2359

Similar to many of the efforts listed above, the parameters included in this checklist are **not** intended to be definitive requirements; instead they are intended as 'points to be considered', with authors themselves deciding which parameters are—and which are not—appropriate for their specific study.

This document is intended to be a living document, which we propose is revisited and amended annually by interested members of the community, who are encouraged to contact the authors of this document. Parts of this document were developed at the annual International Nanomedicine Conference in Sydney, Australia: http://www.oznanomed.org/, which will continue to act as a venue for their review and development, and interested members of the community are encouraged to attend.

After filling out the following pages, this checklist document can be attached as a "Supporting Information" document during submission of a manuscript to inform Editors and Reviewers (and eventually readers) that all points of MIRIBEL have been considered.

Supplementary Table 1. Material characterization*

Question	Yes	No
1.1 Are "**best reporting practices**" **available** for the nanomaterial used? For examples, see *Chem. Mater.* **28** (2016) 3535; http://doi.org/10.1021/acs.chemmater.6b01854 and *Chem. Mater.* **29** (2017) 1; http://doi.org/10.1021/acs.chemmater.6b05235	**Not applicable**	
1.2 If they are available, **are they used**? If not available, ignore this question and proceed to the next one.		
1.3 Are extensive and clear instructions reported detailing all steps of **synthesis** and the resulting **composition** of the nanomaterial? For examples, see *Chem. Mater.* **26** (2014) 1765; http://doi.org/10.1021/cm500632c, and *Chem. Mater.* **26** (2014) 2211; http://doi.org/10.1021/cm5010449. Extensive use of photos, images, and videos are strongly encouraged. For example, see *Chem. Mater.* **28** (2016) 8441; http://doi.org/10.1021/acs.chemmater.6b04639	√	
1.4 Is the **size** (or **dimensions**, if non-spherical) and **shape of** the nanomaterial reported?	√	
1.5 Is the **size dispersity** or **aggregation** of the nanomaterial reported?	√	
1.6 Is the **zeta potential** of the nanomaterial reported?	√	
1.7 Is the **density (mass/volume)** of the nanomaterial reported?	**Not applicable**	
1.8 Is the amount of any **drug loaded** reported? 'Drug' here broadly refers to functional cargos (e.g., proteins, small molecules, nucleic acids).	√	
1.9 Is the **targeting performance** of the nanomaterial reported, including **amount** of ligand bound to the nanomaterial if the material has been functionalised through addition of targeting ligands?	**Not applicable**	
1.10 Is the **label signal** per nanomaterial/particle reported? For example, fluorescence signal per particle for fluorescently labelled nanomaterials.	√	
1.11 If a material property not listed here is varied, has it been **quantified**?	√	
1.12 Were characterizations performed in a **fluid mimicking biological conditions**?	**Not applicable**	
1.13 Are details of how these parameters were **measured/estimated** provided?	√	
Explanation for **No** (if needed):		

*Ideally, material characterization should be performed in the same biological environment as that in which the study will be conducted. For example, for cell culture studies with nanoparticles, characterization steps would ideally be performed on nanoparticles dispersed in cell culture media. If this is not possible, then characteristics of the dispersant used (e.g., pH, ionic strength) should mimic as much as possible the biological environment being studied.

Supplementary Table 2. Biological characterization*

Question	Yes	No
2.1 Are **cell seeding details**, including **number of cells plated, confluency at start of experiment,** and **time between seeding and experiment** reported?	√	
2.2 If a standardised cell line is used, are the **designation and source** provided?	√	
2.3 Is the **passage number** (total number of times a cell culture has been subcultured) known and reported?	**Not applicable**	
2.4 Is the last instance of **verification of cell line** reported? If no verification has been performed, is the time passed and passage number since acquisition from trusted source (e.g., ATCC or ECACC) reported? For information, see *Science* **347** (2015) 938; http://doi.org/10.1126/science.347.6225.938	**Not applicable**	
2.5 Are the results from **mycoplasma testing** of cell cultures reported?	**Not applicable**	
2.6 Is the **background signal of cells/tissue** reported? (E.g., the fluorescence signal of cells without particles in the case of a flow cytometry experiment.)	√	
2.7 Are **toxicity studies** provided to demonstrate that the material has the expected toxicity, and that the experimental protocol followed does not?	√	
2.8 Are details of media preparation (**type of media, serum,** any **added antibiotics**) provided?	√	
2.9 Is a **justification of the biological model** used provided? For examples for cancer models, see *Cancer Res.* **75** (2015) 4016; http://doi.org/10.1158/0008-5472.CAN-15-1558, and *Mol. Ther.* **20** (2012) 882; http://doi.org/10.1038/mt.2012.73, and *ACS Nano* **11** (2017) 9594; http://doi.org/10.1021/acsnano.7b04855	**Not applicable**	
2.10 Is characterization of the **biological fluid** (*ex vivo*/*in vitro*) reported? For example, when investigating protein adsorption onto nanoparticles dispersed in blood serum, pertinent aspects of the blood serum should be characterised (e.g., protein concentrations and differences between donors used in study).	**Not applicable**	
2.11 For **animal experiments**, are the ARRIVE guidelines followed? For details, see *PLOS Biol.* **8** (2010) e1000412; http://doi.org/10.1371/journal.pbio.1000412	√	
Explanation for **No** (if needed):		

*For *in vitro* experiments (e.g., cell culture), *ex vivo* experiments (e.g., in blood samples), and *in vivo* experiments (e.g., animal models). The questions above that are appropriate depend on the type of experiment conducted.

Supplementary Table 3. Experimental details*

Question	Yes	No
3.1 For cell culture experiments: are **cell culture dimensions** including **type of well**, **volume of added media**, reported? Are cell types (i.e.; adherent vs suspension) and **orientation** (if non-standard) reported?	√	
3.2 Is the **dose of material administered** reported? This is typically provided in nanomaterial mass, volume, number, or surface area added. Is sufficient information reported so that regardless of which one is provided, the other dosage metrics can be calculated (i.e. using the dimensions and density of the nanomaterial)?	√	
3.3 For each type of imaging performed, are details of how **imaging** was performed provided, including details of **shielding, non-uniform image processing**, and any **contrast agents** added?	**Not applicable**	
3.4 Are details of how the dose was administered provided, including **method of administration, injection location, rate of administration,** and details of **multiple injections**?	√	
3.5 Is the methodology used to **equalise dosage** provided?	√	
3.6 Is the **delivered dose** to tissues and/or organs (in vivo) reported, as % injected dose per gram of tissue (%ID g^{-1})?	**Not applicable**	
3.7 Is **mass of each organ/tissue measured** and **mass of material** reported?	**Not applicable**	
3.8 Are the **signals of cells/tissues with nanomaterials** reported? For instance, for fluorescently labelled nanoparticles, the total number of particles per cell or the fluorescence intensity of particles + cells, at each assessed timepoint.	√	
3.9 Are **data analysis details**, including **code used** for analysis provided?	√	
3.10 Is the **raw data** or **distribution of values** underlying the reported results provided? For examples, see *R. Soc. Open Sci.* **3** (2016) 150547; http://doi.org/10.1098/rsos.150547, https://opennessinitiative.org/making-your-data-public/, http://journals.plos.org/plosone/s/data-availability, and https://www.nature.com/sdata/policies/repositories	**Not applicable**	
Explanation for **No** (if needed):		

* The use of protocol repositories (e.g., *Protocol Exchange* http://www.nature.com/protocolexchange/) and published standard methods and protocols (e.g., *Chem. Mater.* **29** (2017) 1; http://doi.org/10.1021/acs.chemmater.6b05235, and *Chem. Mater.* **29** (2017) 475; http://doi.org/10.1021/acs.chemmater.6b05481) are encouraged.

References

[1] M. Faria, M. Björnmalm, K. J. Thurecht, S. J. Kent, R. G. Parton, M. Kavallaris, A. P. Johnston, J. J. Gooding, S. R. Corrie, B. J. Boyd, *Nat. Nanotechnol.* **2018**, *13*, 777-785.
